# Supplementary material for: Associations of Microbial Diversity with Age and Other Clinical Variables among Pediatric Chronic Rhinosinusitis (CRS) Patients
Source: Microorganisms. 2023 Feb 7;11(2):422. doi: 10.3390/microorganisms11020422 (PMC9965780; doi:10.3390/microorganisms11020422)
Supplement: Supplementary file 1 [file microorganisms-11-00422-s001.zip › Figure S1_richness_age.pdf]

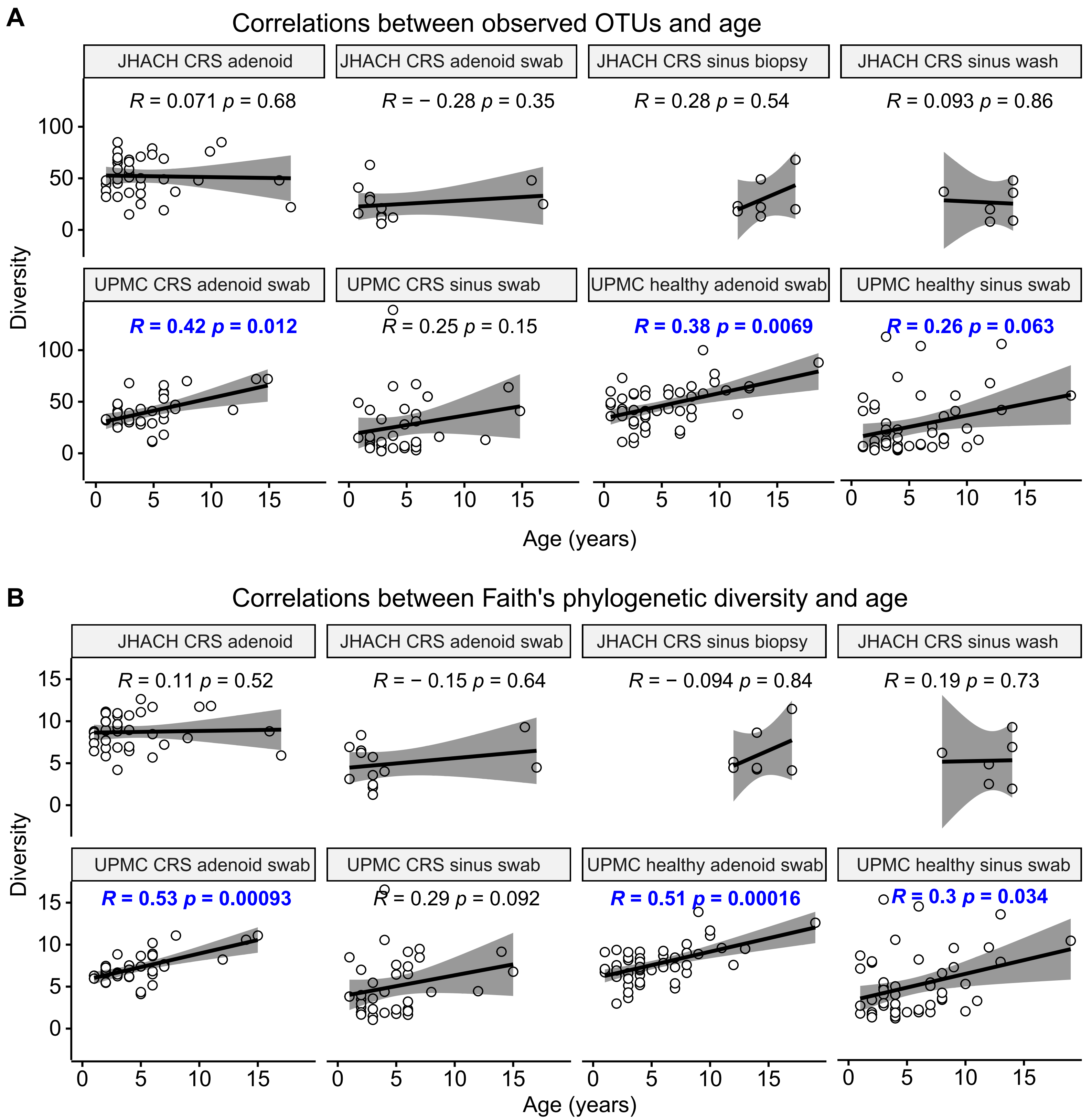
A
Correlations between observed OTUs and age
JHACH CRS adenoid
JHACH CRS adenoid swab
JHACH CRS sinus biopsy
JHACH CRS sinus wash
R = 0.071 p = 0.68
R = − 0.28 p = 0.35
R = 0.28 p = 0.54
R = 0.093 p = 0.86
UPMC CRS adenoid swab
UPMC CRS sinus swab
UPMC healthy adenoid swab
UPMC healthy sinus swab
R = 0.42 p = 0.012
R = 0.25 p = 0.15
R = 0.38 p = 0.0069
R = 0.26 p = 0.063
Diversity
Age (years)
B
Correlations between Faith's phylogenetic diversity and age
JHACH CRS adenoid
JHACH CRS adenoid swab
JHACH CRS sinus biopsy
JHACH CRS sinus wash
R = 0.11 p = 0.52
R = − 0.15 p = 0.64
R = − 0.094 p = 0.84
R = 0.19 p = 0.73
UPMC CRS adenoid swab
UPMC CRS sinus swab
UPMC healthy adenoid swab
UPMC healthy sinus swab
R = 0.53 p = 0.00093
R = 0.29 p = 0.092
R = 0.51 p = 0.00016
R = 0.3 p = 0.034
Diversity
Age (years)
