# Supplementary material for: Associations of Microbial Diversity with Age and Other Clinical Variables among Pediatric Chronic Rhinosinusitis (CRS) Patients
Source: Microorganisms. 2023 Feb 7;11(2):422. doi: 10.3390/microorganisms11020422 (PMC9965780; doi:10.3390/microorganisms11020422)
Supplement: Supplementary file 1 [file microorganisms-11-00422-s001.zip › Table S1.pdf]

Table S1. Metadata of each patient undergoing adenoidectomy and functional endoscopic sinus surgery (FESS) in the Johns Hopkins All Children's Hospital **(**JHACH**)** cohort.

Participant ID	Surgery	Sex	Age (Years)	Sinus culture	Sample type	Included in analysis
ADN001	Adenoidectomy	Female	2	Yes (3 days post-surgery)	Adenoid	Yes
					Adenoid swab	Yes
ADN002	Adenoidectomy	Female	5	No	Adenoid	Yes
ADN003	Adenoidectomy	Male	6	No	Adenoid	Yes
ADN004	Adenoidectomy	Female	3	Yes (~6 months post-surgery)	Adenoid	Yes
ADN005	Adenoidectomy	Male	4	No	Adenoid	Yes
ADN006	Adenoidectomy	Male	11	No	Adenoid	Yes
ADN007	Adenoidectomy	Female	2	No	Adenoid	Yes
ADN008	Adenoidectomy	Male	1	No	Adenoid	Yes
ADN009	Adenoidectomy	Male	2	No	Adenoid	Yes
ADN010	Adenoidectomy	Male	6	No	Adenoid	Yes
ADN011	Adenoidectomy	Female	1	Yes (day of surgery)	Adenoid	Yes
					Adenoid swab	Yes
ADN012	Adenoidectomy	Female	5	No	Adenoid	Yes
ADN013	Adenoidectomy	Male	4	No	Adenoid	Yes
ADN014	Adenoidectomy	Male	17	No	Adenoid	Yes
					Adenoid swab	Yes
ADN015	Adenoidectomy	Male	1	Yes (day of surgery)	Adenoid	Yes
					Adenoid swab	Yes
ADN016	Adenoidectomy	Male	1	Yes (day of surgery)	Adenoid	Yes
ADN017	Adenoidectomy	Female	2	No	Adenoid	Yes
ADN018	Adenoidectomy	Male	2	No	Adenoid	Yes
ADN019	Adenoidectomy	Male	4	No	Adenoid	Yes
ADN020	Adenoidectomy	Female	2	No	Adenoid	Yes
ADN021	Adenoidectomy	Male	3	Yes (day of surgery)	Adenoid	No
					Adenoid swab	Yes
ADN022	Adenoidectomy	Male	4	No	Adenoid	Yes
ADN023	Adenoidectomy	Male	6	No	Adenoid	Yes
ADN024	Adenoidectomy	Male	1	No	Adenoid	Yes
ADN025	Adenoidectomy	Male	16	Yes (day of surgery)	Adenoid	Yes
					Adenoid swab	Yes
ADN026	Adenoidectomy	Male	5	No	Adenoid	No
ADN027	Adenoidectomy	Male	3	Yes (day of surgery)	Adenoid	Yes
					Adenoid swab	Yes

ADN028	Adenoidectomy	Male	3	Yes (day of surgery)	Adenoid	Yes
					Adenoid swab	Yes
ADN029	Adenoidectomy	Female	2	Yes (day of surgery)	Adenoid	Yes
					Adenoid swab	Yes
ADN030	Adenoidectomy	Female	2	Yes (day of surgery)	Adenoid	Yes
					Adenoid swab	Yes
ADN031	Adenoidectomy	Male	3	Yes (day of surgery)	Adenoid	Yes
					Adenoid swab	Yes
ADN032	Adenoidectomy	Male	7	No	Adenoid	Yes
ADN033	Adenoidectomy	Female	9	No	Adenoid	Yes
ADN034	Adenoidectomy	Female	3	No	Adenoid	Yes
ADN035	Adenoidectomy	Female	3	Yes (day of surgery)	Adenoid	Yes
					Adenoid swab	Yes
ADN036	Adenoidectomy	Male	2	Yes (day of surgery)	Adenoid	No
					Adenoid swab	No
ADN037	Adenoidectomy	Male	4	Yes (day of surgery)	Adenoid	Yes
					Adenoid swab	Yes
ADN038	Adenoidectomy	Female	10	No	Adenoid	Yes
ADN039	Adenoidectomy	Male	3	No	Adenoid	Yes
ADN040	Adenoidectomy	Male	2	No	Adenoid	Yes
CRS_C001	FESS	Female	14	Yes (day of surgery)	Sinus	Yes
					Sinus wash (left maxillary)	Yes
					Sinus wash (right maxillary)	Yes
CRS_C002	FESS	Female	8	Yes (day of surgery)	Sinus	Yes
					Sinus wash	Yes
CRS_C004	FESS	Female	14	Yes (day of surgery)	Sinus (left maxillary)	Yes
					Sinus (right maxillary)	Yes
					Sinus wash (left maxillary)	No
					Sinus wash (right maxillary)	Yes
CRS_C005	FESS	Male	12	Yes (day of surgery)	Sinus (left maxillary)	Yes
					Sinus (right maxillary)	Yes
					Sinus wash (left maxillary)	Yes
					Sinus wash (right maxillary)	Yes
CRS_C006	FESS	Male	17	Yes (day of surgery)	Sinus (left maxillary)	Yes
					Sinus (right maxillary)	Yes
					Sinus wash (left maxillary)	No
					Sinus wash (right maxillary)	No
