# Supplementary material for: Associations of Microbial Diversity with Age and Other Clinical Variables among Pediatric Chronic Rhinosinusitis (CRS) Patients
Source: Microorganisms. 2023 Feb 7;11(2):422. doi: 10.3390/microorganisms11020422 (PMC9965780; doi:10.3390/microorganisms11020422)
Supplement: Supplementary file 1 [file microorganisms-11-00422-s001.zip › Table S2.pdf]

Table S2. Adjusted p-values for multiple pairwise Mann-Whitney-U comparisons of alpha diversity metrics between sample subgroups from chronic rhinosinusitis (CRS) patients, healthy controls, and technical controls in the Johns Hopkins All Children's Hospital (JHACH) and/or University of Pittsburgh Medical Center (UPMC) cohorts. Alpha diversity metrics included A) Faith's phylogenetic diversity, B) observed operational taxonomic units (OTUs), C) Shannon diversity, and D) Pielou's evenness. Statistically significant differences ($p<0.05$) are indicated in bold.

A. Faith's phylogenetic diversity	JHACH: adenoid	JHACH: adenoid swab	JHACH: sinus biopsy	JHACH: sinus wash	UPMC: adenoid swab	UPMC: sinus swab	UPMC: Healthy adenoid swab	UPMC: Healthy sinus swab	UPMC: Extraction kit	UPMC: PCR reagents
JHACH: adenoid swab (CRS)	**0.0003**	-	-	-	-	-	-	-	-	-
JHACH: sinus biopsy (CRS)	**0.0398**	0.5600	-	-	-	-	-	-	-	-
JHACH: sinus wash (CRS)	**0.0270**	0.8966	0.9563	-	-	-	-	-	-	-
UPMC: adenoid swab (CRS)	**0.0114**	**0.0114**	0.1450	0.1650	-	-	-	-	-	-
UPMC: sinus swab (CRS)	**0.0000**	0.8596	0.3281	0.7805	**0.0044**	-	-	-	-	-
UPMC: adenoid swab (control)	0.0788	**0.0048**	0.1068	0.0848	0.3510	**0.0005**	-	-	-	-
UPMC: sinus swab (control)	**0.0000**	0.7805	0.2826	0.7568	**0.0008**	0.9563	**0.0001**	-	-	-
UPMC: extraction kit	**0.0085**	0.9563	0.5397	0.9563	**0.0436**	0.8867	**0.0398**	0.8350	-	-
UPMC: PCR reagents	0.1068	0.4029	0.3716	0.4029	0.1068	0.4583	0.1650	0.3281	0.5366	-
UPMC: Zymo reagents	**0.0065**	**0.0398**	**0.0345**	**0.0398**	**0.0065**	**0.0638**	**0.0062**	**0.0398**	**0.0634**	0.8105

B. Observed OTUs	JHACH: adenoid	JHACH: adenoid swab	JHACH: sinus biopsy	JHACH: sinus wash	UPMC: adenoid swab	UPMC: sinus swab	UPMC: Healthy adenoid swab	UPMC: Healthy sinus swab	UPMC: Extraction kit	UPMC: PCR reagents
JHACH: adenoid swab (CRS)	**0.0012**	-	-	-	-	-	-	-	-	-
JHACH: sinus biopsy (CRS)	**0.0469**	0.6377	-	-	-	-	-	-	-	-
JHACH: sinus wash (CRS)	**0.0254**	0.8807	0.7596	-	-	-	-	-	-	-
UPMC: adenoid swab (CRS)	**0.0246**	**0.0246**	0.2008	0.2055	-	-	-	-	-	-
UPMC: sinus swab (CRS)	**0.0001**	0.7398	0.3131	0.7398	0.0064	-	-	-	-	-
UPMC: adenoid swab (control)	0.2109	**0.0064**	0.1307	0.0602	0.2948	**0.0005**	-	-	-	-
UPMC: sinus swab (control)	**0.0000**	0.6108	0.3357	0.6452	**0.0012**	0.9004	**0.0001**	-	-	-
UPMC: extraction kit	**0.0183**	0.8807	0.6452	0.8207	**0.0493**	0.7596	**0.0325**	0.7596	-	-
UPMC: PCR reagents	0.2020	0.4513	0.3819	0.8807	0.2020	0.7596	0.2020	0.8755	0.4084	-
UPMC: Zymo reagents	**0.0064**	0.1168	**0.0325**	0.1391	**0.0064**	0.3684	**0.0064**	0.4084	**0.0670**	0.3704

C. Shannon Index	JHACH: adenoid	JHACH: adenoid swab	JHACH: sinus biopsy	JHACH: sinus wash	UPMC: adenoid swab	UPMC: sinus swab	UPMC: Healthy adenoid swab	UPMC: Healthy sinus swab	UPMC: Extraction kit	UPMC: PCR reagents
JHACH: adenoid swab (CRS)	**0.0001**	-	-	-	-	-	-	-	-	-
JHACH: sinus biopsy (CRS)	0.6388	**0.0218**	-	-	-	-	-	-	-	-
JHACH: sinus wash (CRS)	0.4068	0.1873	0.9012	-	-	-	-	-	-	-
UPMC: adenoid swab (CRS)	**0.0039**	**0.0072**	0.2510	0.7037	-	-	-	-	-	-

UPMC: sinus swab (CRS)	**0.0000**	0.4068	**0.0052**	**0.0186**	**0.0000**	-	-	-	-	-
UPMC: adenoid swab (control)	0.1041	**0.0039**	0.6987	1.0000	0.3748	**0.0000**	-	-	-	-
UPMC: sinus swab (control)	**0.0000**	0.4785	**0.0039**	**0.0204**	**0.0000**	0.8041	**0.0000**	-	-	-
UPMC: extraction kit	0.2510	0.4785	0.4904	0.7002	0.6608	0.2064	0.4904	0.2127	-	-
UPMC: PCR reagents	0.4523	0.6984	0.4436	0.9066	0.7002	0.6944	0.4904	0.5197	1.0000	-
UPMC: Zymo reagents	**0.0458**	0.1225	**0.0218**	0.6388	0.6608	0.2625	0.2707	0.1041	1.0000	0.5641

D. Pielou's evenness	JHACH: adenoid	JHACH: adenoid swab	JHACH: sinus biopsy	JHACH: sinus wash	UPMC: adenoid swab	UPMC: sinus swab	UPMC: Healthy adenoid swab	UPMC: Healthy sinus swab	UPMC: Extraction kit	UPMC: PCR reagents
JHACH: adenoid swab (CRS)	**0.0002**	-	-	-	-	-	-	-	-	-
JHACH: sinus biopsy (CRS)	0.0553	**0.0027**	-	-	-	-	-	-	-	-
JHACH: sinus wash (CRS)	0.3933	**0.0113**	0.5251	-	-	-	-	-	-	-
UPMC: adenoid swab (CRS)	**0.0033**	**0.0239**	**0.0012**	**0.0133**	-	-	-	-	-	-
UPMC: sinus swab (CRS)	**0.0000**	0.5251	**0.0002**	0.0025	**0.0010**	-	-	-	-	-
UPMC: adenoid swab (control)	0.0553	**0.0085**	0.0082	0.0899	0.2582	**0.0001**	-	-	-	-
UPMC: sinus swab (control)	**0.0000**	0.5361	**0.0003**	**0.0010**	**0.0001**	0.8462	**0.0000**	-	-	-
UPMC: extraction kit	1.0000	0.1554	0.4127	0.6705	0.5082	0.0557	0.5361	0.0726	-	-
UPMC: PCR reagents	0.5146	0.2086	1.0000	0.8895	0.2350	0.0899	0.2086	0.1509	0.8462	-
UPMC: Zymo reagents	**0.0002**	**0.0027**	**0.0133**	**0.0202**	**0.0002**	**0.0002**	**0.0029**	**0.0029**	**0.0553**	0.5082
