# Supplementary material for: Associations of Microbial Diversity with Age and Other Clinical Variables among Pediatric Chronic Rhinosinusitis (CRS) Patients
Source: Microorganisms. 2023 Feb 7;11(2):422. doi: 10.3390/microorganisms11020422 (PMC9965780; doi:10.3390/microorganisms11020422)
Supplement: Supplementary file 1 [file microorganisms-11-00422-s001.zip › Table S3.pdf]

Table S3. Genera significantly correlated with age in adenoid-derived samples.

Genus	p value	Spearman's ρ
**Subgroup: JHACH adenoid (CRS)**		
Alloprevotella	0.00	-0.47
Bergeyella	0.01	-0.42
Candidatus Saccharimonas	0.04	0.34
Capnocytophaga	0.00	-0.49
Dialister	0.00	0.46
Eubacterium brachy group	0.00	0.50
Granulicatella	0.01	-0.44
Haemophilus	0.03	-0.35
Johnsonella	0.00	-0.58
F0058	0.05	0.33
Slackia	0.02	0.37
Sphingomonas	0.05	0.33
Streptobacillus	0.00	-0.47
Treponema	0.03	0.36
**Subgroup: JHACH adenoid swab (CRS)**		
Burkholderia-Caballeronia-Paraburkholderia	0.04	0.56
Leptotrichia	0.02	-0.65
Neisseria	0.00	-0.86
Streptobacillus	0.03	-0.60
**Subgroup: UPMC adenoid swab (CRS)**		
Abiotrophia	0.03	0.36
Aggregatibacter	0.02	0.40
Atopobium	0.01	0.42
Dialister	0.02	0.40
Eubacterium nodatum group	0.02	0.39
Johnsonella	0.00	-0.63
Mogibacterium	0.03	0.37
Oribacterium	0.04	0.35
F0058	0.01	0.41
Prevotella	0.03	0.37
Saccharimonadaceae	0.03	0.37
Selenomonas	0.01	0.44
Solobacterium	0.00	0.51
Stomatobaculum	0.03	0.36
Streptobacillus	0.00	-0.50
Treponema	0.03	0.37
**Subgroup: UPMC adenoid swab (control)**		
Absconditabacteriales (SR1)	0.00	0.40
Actinomyces	0.03	0.30
Atopobium	0.04	0.29
Bacilli RF39	0.00	0.41
Candidatus Saccharimonas	0.02	0.32
Dialister	0.00	0.42
Eubacterium nodatum group	0.01	0.39
Johnsonella	0.00	-0.42
Lentimicrobium	0.05	0.28
Megasphaera	0.01	0.35
Mogibacterium	0.00	0.47
Mycoplasma	0.01	0.35
Oribacterium	0.00	0.42
Peptostreptococcus	0.00	0.43

Prevotella	0.03	0.32
TM7x	0.01	0.35
Solobacterium	0.00	0.45
Streptobacillus	0.00	-0.43
Tannerella	0.00	0.49
