# Supplementary material for: Associations of Microbial Diversity with Age and Other Clinical Variables among Pediatric Chronic Rhinosinusitis (CRS) Patients
Source: Microorganisms. 2023 Feb 7;11(2):422. doi: 10.3390/microorganisms11020422 (PMC9965780; doi:10.3390/microorganisms11020422)
Supplement: Supplementary file 1 [file microorganisms-11-00422-s001.zip › Table S4.pdf]

Table S4. Genera significantly correlated with age in sinus-derived samples.

Genus	p value	Spearman's ρ
**Subgroup: JHACH sinus (CRS)**		
Corynebacterium	0.05	-0.76
Dolosigranulum	0.02	-0.82
Eubacterium coprostanoligenes group	0.02	0.82
Staphylococcus	0.03	0.81
**Subgroup: UPMC sinus swab (CRS)**		
Anaerococcus	0.04	0.35
Capnocytophaga	0.04	-0.35
Dialister	0.01	0.42
Kocuria	0.04	-0.36
Lawsonella	0.00	0.61
Peptoniphilus	0.04	0.34
Staphylococcus	0.00	0.54
Tepidimonas	0.03	0.36
Treponema	0.02	0.38
**Subgroup: UPMC sinus swab (control)**		
Absconditabacteriales (SR1)	0.00	0.41
Actinobacillus	0.02	0.33
Actinomyces	0.04	0.30
Aggregatibacter	0.00	0.43
Campylobacter	0.00	0.42
UCG-014	0.00	0.40
Delftia	0.00	0.40
Dialister	0.02	0.32
Eikenella	0.03	0.31
Fusobacterium	0.01	0.35
Johnsonella	0.02	0.33
Lentimicrobium	0.04	0.29
Mogibacterium	0.04	0.28
Neisseria	0.01	0.39
Oribacterium	0.01	0.38
Parvimonas	0.00	0.44
Peptostreptococcus	0.02	0.34
Ralstonia	0.04	-0.29
Rodentibacter	0.02	0.32
Staphylococcus	0.00	0.54
Tannerella	0.02	0.32
