# Supplementary figures and images for: Associations of Microbial Diversity with Age and Other Clinical Variables among Pediatric Chronic Rhinosinusitis (CRS) Patients
Source: Microorganisms. 2023 Feb 7;11(2):422. doi: 10.3390/microorganisms11020422 (PMC9965780; doi:10.3390/microorganisms11020422)

# Differentially abundant genera between JHACH sinus tissue and sinus wash samples

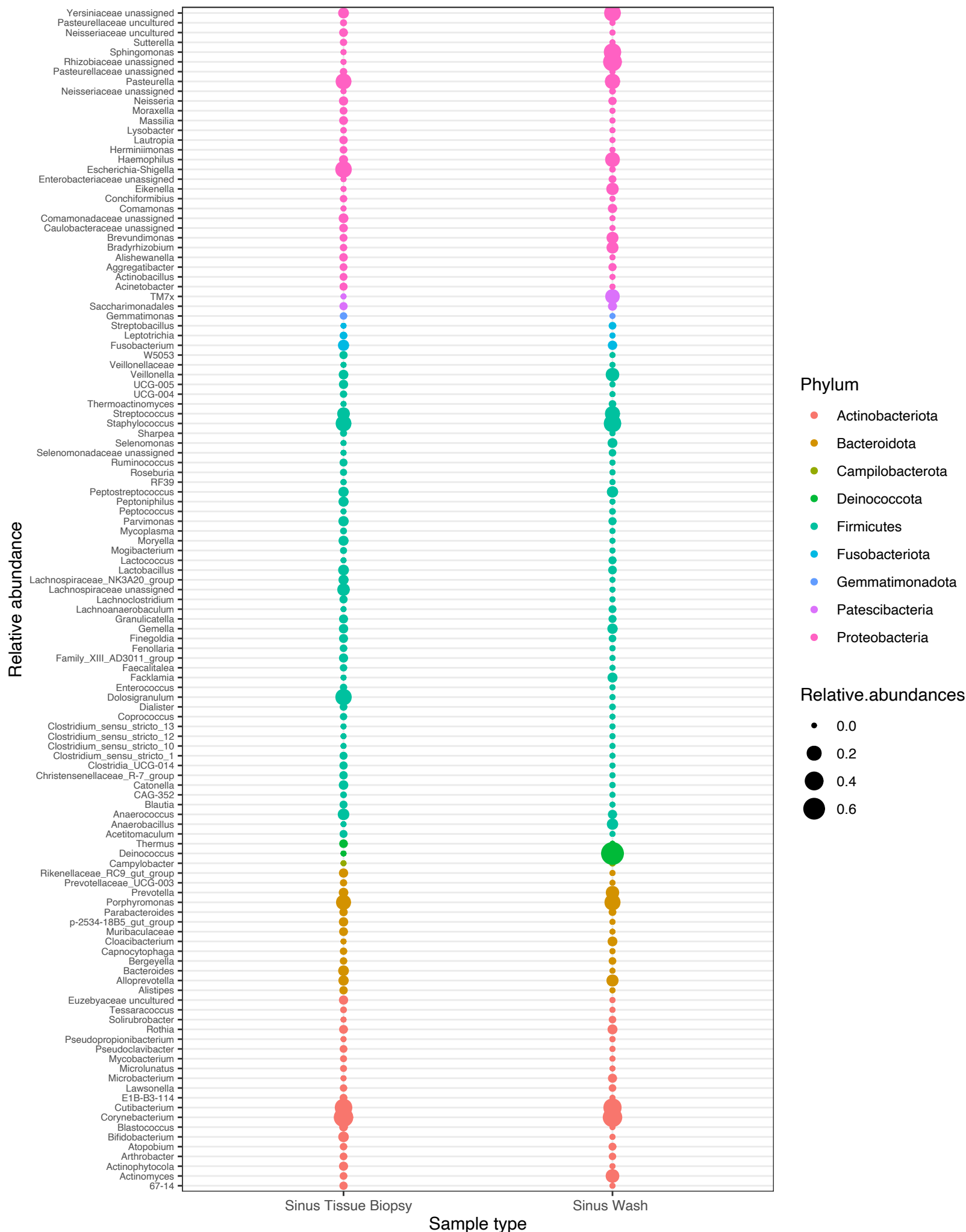

Supplement: Supplementary file 1 [file microorganisms-11-00422-s001.zip › Figure S2_ANCOM_JHACH_Sinus.pdf]
